# More efficient, smaller multicancer screening trials

**DOI:** 10.1093/jnci/djae251

**Published:** 2024-10-14

**Authors:** Peter Sasieni, Adam R Brentnall

**Affiliations:** Wolfson Institute of Population Health, Queen Mary University of London, London, United Kingdom; Wolfson Institute of Population Health, Queen Mary University of London, London, United Kingdom

## Abstract

**Background:**

The NHS-Galleri Trial has demonstrated feasibility of a trial design in which all participants provide a “sample” for screening, but only samples from the intervention arm are tested and acted upon during the trial. We assessed the efficiency of analysis methods when the control arm may be retrospectively tested at the time of analysis.

**Methods:**

Analyses considered were (1) the traditional method (random allocation, with all events included), (2) the “intended-effect” method (nested in those individuals who tested positive in both arms and all events therein), and (3) the targeted method (by random allocation but with an endpoint “test-positive event”). These methods are compared using approximate statistical methods and scenario analysis.

**Results:**

Provided that the number of individuals who die from cancer after a test-positive sample is a small fraction of the total number who die from cancer, intended-effect and targeted analyses require a much smaller sample size to evaluate cancer-specific mortality than the traditional approach. Intended-effect analysis has a smaller sample size requirement than targeted analysis does. This gain is substantial only when the risk of cancer death in individuals testing positive is high.

**Conclusion:**

Intended-effect or targeted analysis may substantially reduce the sample size needed to evaluate cancer-specific mortality in blood-based screening trials. Targeted analysis requires many fewer retrospective tests and avoids potential problems arising from the need to inform those individuals whose stored samples test positive. Trialists should consider the trade-off of costs between sample size and retrospective testing requirements when choosing the analysis method.

## Introduction

**Figure 1.**
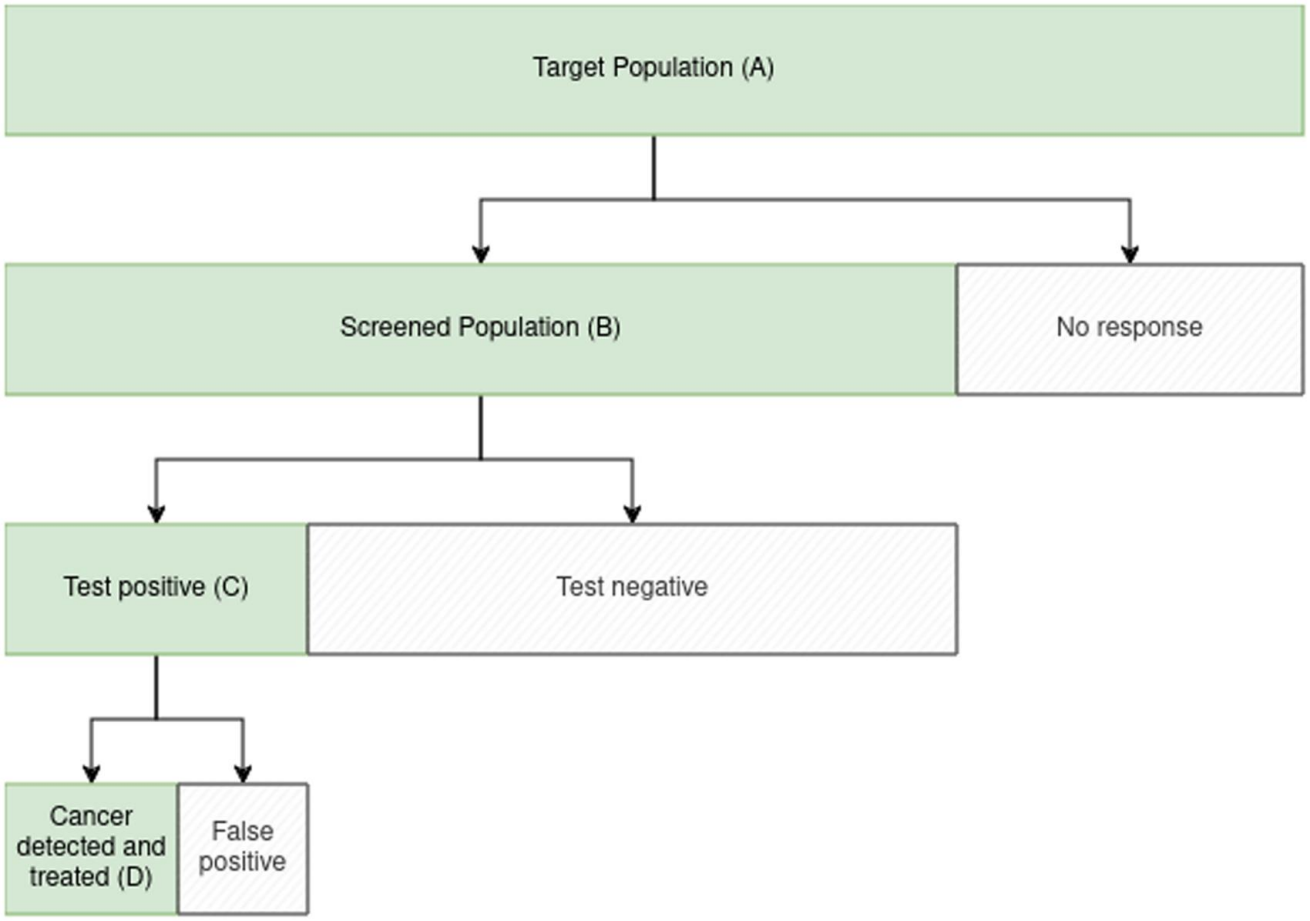
Population groups in screening trials. The bar widths, which are not to scale, indicate the population size.

A framework to discuss analysis methods for cancer screening trials is introduced in Figure 1. Here, screening is divided into four sequential components: approach to be screened, testing, triage of positive tests, and treatment of screen-detected disease. Screening is offered to population A, but it can benefit only a sub-population B, the members of which accept the offer and receive the screening test. The test identifies a subset of individuals screened for whom investigations or treatment are recommended. Any benefit is further restricted to individuals who test positive (population C). They are typically triaged with additional tests. Triage may use a reflex test based on material from the screening sample (eg, cervical screening samples positive for human papillomavirus are processed for liquid-based cytology) or require an additional clinical visit (eg, colonoscopy for individuals with a positive fecal occult blood test finding). Where feasible, reflex testing avoids loss to follow-up and needlessly worrying people with the result of the first test. Individuals routed through triage subsequently undergo a reference test when a definitive diagnosis is made, and then “treatment” is offered to individuals with detected disease. This treatment may be a combination of medical (or surgical) management of clinically-significant disease, active surveillance of indolent cancer, or more intensive screening for individuals not found to have cancer but for whom the risk remains elevated. The aim of screening is to identify and treat cancer and initiate treatment early in patients with undiagnosed, nonindolent disease (population D).

This framework makes it clear how “noise” may swamp the “signal” in screening trials. In terms of efficacy, the only people who may benefit from screening are in population D (ie, individuals who have their cancer treated early). This group is much smaller than the group tested or approached. Even in population D cancer mortality may occur at a low rate and take many years to accumulate before analysis can be done. Different harms are restricted to subsets of individuals invited. Typically, there is no harm from the invitation per se. The people tested (population B) have whatever harms (pain, exposure to radiation, etc) are caused by the screening test, but most of the serious harms are restricted to individuals who test positive (population C). People with false-positive test results may be made anxious or undergo invasive investigations. People who have screen-detected cancer may be overtreated if they would not have been diagnosed with cancer in their lifetime but for the screening test.

Random assignment is used in screening trials to evaluate the causal effect of screening. Designs with randomization in population A do not efficiently answer the causal inference question.^1^ We therefore focus on trials designed to estimate the effect of a screening intervention on individuals who engage with it (population B)—that is, efficacy.^1^ In this article, we consider sample-size requirements for such trials where samples are taken and stored in both the intervention and the control arms.

## Methods

### Designs

To ensure complete sample collection in both arms at baseline, random assignment ideally takes place after a participant has provided their first sample. Only samples from the intervention group are tested and acted upon during the trial. We primarily consider designs using either a single round of screening or blinding to random assignment so that repeated samples are collected with the same retention in both arms.

#### Intended-effect analysis (analysis restricted to individuals who test positive)

One can test all samples at the end of the trial and analyze the data as if one had only randomly assigned people with a positive sample (ie, population C). This approach has previously been discussed and called an “intended-effect” analysis.^2^^,^^3^ The analysis requires testing of all control arm samples.

#### Targeted analysis (event restricted to individuals who test positive)

Alternatively, one might test stored samples only from individuals with the event of interest (eg, death from specific types of cancer). As there is no expectation of an effect of screening on individuals who test negative, this approach investigates whether the intervention reduces the number of test-positive events. The approach was adopted in the *Helicobacter pylori* Screening Study (ISRCTN71557037), which was designed to reduce the incidence of gastric cancer.^4^ It has been proposed for multicancer screening^5^ and is planned for secondary endpoints in the NHS-Galleri trial of a multicancer early detection test for screening.^6^

#### Traditional analysis (all events in all randomly assigned individuals)

The traditional analysis evaluates cancer-specific mortality by random assignment. This approach requires no further testing of stored samples. It is most efficient when random assignment is in population C (ie, individuals with a positive test are randomly assigned between reveal and conceal), but such trials are rare. It is the standard approach when randomization is in population A or B.

### Statistical analysis

We next consider the mean and variance of the relevant test statistics to gain insight into the magnitude of the impact of different analysis methods on power and sample size. To that end, we used three main assumptions.

- No effect of testing on outcomes in individuals who test negative (population B, but not population C)
- No effect of storage on test performance
- No difference in sample provision (in individuals without a previous positive sample) between arms (eg, the probability of returning a sample at visit 3 in someone who has not had a previous sample that would have been positive if tested is independent of random assignment)

####  

We considered testing using a χ^2^ test of proportions, which has comparable sample size requirements with a log-rank test if the event rate is small, as expected for cancer-specific mortality in a screening trial.^7^ With 1:1 random assignment, denominators for targeted analysis are identical in both arms (number randomly assigned). Random assignment also ensures identical expected denominators for intended-effect analysis in both arms (expected number of individuals who test positive). It follows that most information about efficacy is from the shared numerator (number of events in individuals who test positive). Therefore, it may be sufficient to retrospectively test control individuals with an event (targeted analysis). We identified that design differences in power for a trial with n individuals per arm are driven by the following considerations (see Supplementary Material for more general formulae).

First, the traditional approach contrasts the number of events *r_j_* in each arm *j *=* *0, 1 (control, screening) through $\left(r_{1}-r_{0}\right)$. Events may be decomposed into test positive *r_pj_* or negative *r_nj_*, and (with rare events) variance is approximately $\left(r_{p1}+r_{n1}+r_{p0}+r_{n0}\right)$. Using the assumption of no effect in individuals who test negative, the targeted test statistic $\left(r_{p1}-r_{p0}\right)$ has the same expectation as the traditional statistic but with a smaller variance—that is, $\left(r_{p1}+r_{p0}\right)$. The variance is reduced by $\left(r_{n1}+r_{n0}\right)$; the more events in individuals who test negative, the greater the reduction in sample size. The factor that determines the benefit of targeted vs traditional designs is the ratio $\left(r_{p0}+r_{p1})/(r_{0}+r_{1}\right)$; the required sample size is multiplied by the proportion of events that are in individuals who also test positive.

Second, recall that the intended-effect analysis is restricted to individuals who test positive. The test statistic is the same as in the targeted analysis, but we can condition on the number of individuals who test positive ($n_{p0},\;n_{p1}$) in each arm. An estimate of the conditional variance of ($r_{p1}-r_{p0}$) is $\left\{r_{p1}(1-r_{p1}/n_{p1})+r_{p0}(1-r_{p0}/n_{p0})\right\}$. Therefore, relative sample size requirements depend on the factors $\left(1-r_{p0}/n_{p0}\right)$ and $\left(1-r_{p1}/n_{p1}\right)$; approximately, the sample size is multiplied by the proportion of test-positive individuals who do not have an event. Intended-effect analysis will offer a marked reduction in sample size over the targeted approach only if events are common in individuals who test positive (at least in the control arm).

### Scenario analysis

To evaluate the potential role of the new methods in trial design, we applied the sample size formulae (Supplementary Material) to scenarios previously reported,^2^^,^^5^ and new scenarios for multicancer early detection trials. The designs were evaluated based on sample size and trial cost.

## Results

### Single-cancer screening

Data from three completed screening trials were previously used to evaluate intended-effect analysis.^2^ Although this analysis would not have been possible in any of these trials (screening samples were not stored for control participants), it usefully demonstrated that the intended-effect analysis could substantially reduce sample size. We revisit the examples in Table 1, including the targeted approach. Results are consistent with standard calculations using statistical software and the previous intended-effect analysis.^2^ The bulk of the gains in smaller sample size from the intended-effect analysis would also be achieved using a targeted approach but with many fewer retrospective tests. There is no substantial gain from intended-effect analysis because event rates are low in individuals who test positive.

**Table 1.** Sample size scenarios for trials to evaluate cancer-specific mortality or overdiagnosis^a^

Scenario		1	2	3	4	5	6
Trial type		Minnesota Colon Cancer Control Study	Prostate, Lung, Colorectal, Ovarian Cancer Screening Trial (colorectal component)	National Lung Cancer Screening Trial	Multicancer early detection	Multicancer early detection	Multicancer early detection
Event for cancer		Death	Death	Death	Death	Death	Incidence

Traditional approach, outcomes							
Events, control, %	*p*_0_	0.78	0.45	1.68	1.08	1.70	3.00
Events, screening, %	*p*_1_	0.53	0.32	1.35	0.92	1.57	3.11
Relative risk	$p_{1}/p_{0}$	0.68	0.72	0.80	0.85	0.93	1.04
Intended effect, outcomes							
Events given positive test, control, %	*ρ_p_*	1.15	0.80	3.49	11.49	19.28	47.95
Events given positive test, screening, %	$\theta \rho_{p}$	0.55	0.35	2.62	8.04	15.43	57.55
Relative risk	*θ*	0.48	0.44	0.75	0.70	0.80	1.20
Targeted, outcomes							
Test-positive events, control, %	$\pi \rho_{p}$	0.48	0.22	1.33	0.54	0.64	0.53
Test-positive events, screening, %	$\pi \rho_{p}\theta$	0.23	0.10	0.99	0.38	0.51	0.63
Other parameters							
Positive test, %	*π*	42.0	28.0	38.0	4.7	3.3	1.1
Events in individuals with a negative test, %	*ρ_n_*	0.51	0.31	0.57	0.57	1.10	2.50
Risk ratio for positive and negative tests	$\rho_{p}/\rho_{n}$	2.25	2.55	6.11	20.28	17.53	19.18
“Ever positive” test summary statistics							
Proportion of events that are positive, %	$\pi \rho_{p}/p_{0}$	61.9	49.8	78.9	50.0	37.4	17.6
Proportion of individuals without an event who are negative, %	$\left(1-\pi )(1-\rho_{n})/(1-p_{0}\right)$	58.2	72.1	62.7	95.8	97.3	99.4
Approximate sample size for 90% power (per arm)							
Traditional	*N*_1_	21 649	51 557	28 514	79 190	208 856	558 784
Targeted	*N*_2_	11 858	21 507	21 925	36 580	73 879	108 915
Intended effect	*N*_3_	11 792	21 404	21 497	33 053	61 255	51 288
Relative size	$N_{1}/N_{2}$	1.83	2.40	1.30	2.16	2.83	5.13
Relative size	$N_{2}/N_{3}$	1.01	1.00	1.02	1.11	1.21	2.12

a The parameters include use of data from 3 completed cancer screening trials.

### Multicancer screening

Hackshaw and Berg^5^ previously recommended analysis of test-positive cancer deaths in multicancer early detection trials (ie, the targeted approach). Reanalysis of their scenario 4 in Table 1 demonstrated a sample size gain from an intended-effect analysis compared with targeted analysis but much less than both achieve over the traditional approach. Note that the intended-effect analysis requires randomly assigning approximately 7000 fewer individuals but testing approximately 33 000 additional control samples per round of screening. A similar finding is observed in scenario 5. Here, efficacy is less than scenario 4, but the event rate is higher. The assumptions reflect a scenario in which the endpoint uses a wider pool of cancers over repeated screens. Scenario 6 considers estimation of overdiagnosis from one screen. The multicancer early detection sensitivity is imperfect; therefore, substantial gains are expected in the targeted vs traditional analysis, as observed. In addition, cancer detection is common in individuals who test positive, so substantial gains are seen from intended effect compared with the targeted approach.

### Efficiency

Estimation using intended-effect analysis yields greater statistical power than the targeted approach, and given a dataset where everyone was tested, it should be used. In scenarios 1 through 3, however, intended-effect analysis does not materially reduce sample size, requires substantially more testing, and will be more expensive. If there are marked gains in reduced sample size, such as in multicancer early detection scenario 5, then targeted analysis might still be preferred if overall it costs less. The cost in scenario 5 of recruiting an additional 25 000 participants should be compared with the cost of testing 60 000 additional samples per round of screening. The NHS-Galleri trial provides a real-world example in which either targeted or intended-effect analysis could be used, but targeted analysis is planned for cancer-specific mortality.^6^ Five years from the last participant randomly assigned, we estimated approximately 1200 cancer deaths in 70 000 individuals randomly assigned to control, which may require testing approximately 2500 samples (3 screening rounds, but some of the cancers will have been diagnosed before the second or third sample collection). By comparison, the intended-effect analysis would require testing approximately 200 000 samples; the current commercial price for the Galleri test is $945 per sample. Recruitment costs depend on the time trial infrastructure is kept running. Recruitment to NHS-Galleri used data-driven dynamic methods and took less than 11 months,^8^^,^^9^ with all three screening rounds completed within 4 years. In other trials where the test costs less or recruitment takes longer and costs more, an intended-effect analysis might offer best value for the money.

## Discussion

Under relatively mild assumptions, both targeted and intended-effect analyses can substantially reduce sample size for cancer screening studies. Before drawing broader conclusions, however, we discuss some limitations of these methods, how drop-out (loss to sampling) may affect analysis, and sample-size calculations.

### Disadvantages of retrospective testing

There are disadvantages to both intended-effect and targeted analysis. One is that test results at the time of analysis must be the same as when the sample was taken. Whether this holds will depend on the stability of the sample for the assay and assay quality control. Collecting a spare aliquot and re-testing a proportion from the intervention arm would help estimate the impact of storage on the results. Another disadvantage is that the cost of biobanking control samples may be substantial. Intended-effect analysis has two additional disadvantages. First, the cost of analyzing all samples may be substantial. Intended-effect analysis requires analysis of all stored samples, but the extra testing over targeted analysis is worthwhile only if many test-positive individuals have the event of interest. Second, the trial must decide whether individuals testing positive retrospectively should be informed. If required, this information could create anxiety and confusion and potentially reduce the ability to study long-term outcomes because of “delayed screening.” However, with a long interval after the sample was collected, many of the retrospective positive tests in individuals without a cancer diagnosis will likely be false positives.

### Adherence to sampling

Adherence to sample collection will affect the sample size and power of the new methods. We have focused on blinded repeat-screen designs, or evaluation of a single screen with random assignment after sample collection. With repeat screening, drop-out is inevitable, but if blinding is maintained, one might expect drop-out to be approximately equal between arms. In this case, sample size calculations can be revised by adjusting the ever test-positive parameter.

If the design does not use blinding or blinding is compromised, then one might expect fewer samples in individuals randomly assigned to the control arm. If the prognosis in individuals who would test positive is independent of sample availability (independent-retention assumption), then intended-effect and targeted approaches can be used. Both methods will be similarly affected by the reduction in power from fewer test-positive events. To maintain consistency and power, the targeted approach must be adjusted for the expected number of positive tests among individuals with an event but no stored samples because fewer samples will lead to fewer positive tests in the control arm. A simple approach to correct for this is to multiply the number of individuals with an event but a missing sample by the proportion of individuals with an event whose sample was positive in the same arm. More generally, one could estimate the probability of a positive result, adjusting for various factors such as using logistic regression. With the independent-retention assumption and such adjustments, the targeted approach is still valid.

If the independent-retention assumption fails, then the new methods are less attractive to evaluate repeat screening. This failure could occur if, for example, older participants (eg, aged >65 y), participants with comorbidities, or participants with other factors associated with prognosis given positive tests are less likely to provide a sample if randomly assigned to the control arm than to the intervention arm. A naive intended-effect analysis would not control type I error. To be conservative, one might apply the targeted analysis without the above corrections, but doing so would lose power and may be less efficient than the traditional approach.

For nonblinded designs, therefore, one may prefer to apply the new methods to the baseline screen only. An extreme scenario is when there are no samples in the control arm after baseline by design, as occurred in the UK Collaborative Trial of Ovarian Cancer Screening.^10^ Depending on assumptions, using the new methods to the baseline screen could still be more powerful than the traditional approach. For instance, trialists might reserve the new methods to evaluate overdiagnosis (scenario F) and a traditional analysis for cancer-specific mortality. Analysis of the baseline screen alone may also be useful for an interim analysis early readout on mortality.

In summary, application of these methods requires a decision on whether to analyze multiple screening rounds, partly because differential sample completion may be a threat to the validity of retrospective testing analysis. Restricting analyses to the earlier rounds may be most useful because these are expected to have the greatest impact within the duration of the trial and the most complete sample data.

### Sample-size calculations

Sample-size calculations for targeted and intended-effect analysis require an additional parameter over the traditional approach. In practice, one is likely to have information about event rates and the test-positivity rate from prior studies. The additional parameter needed is the rate in individuals who test negative or positive, or their ratio, or the screening effect in individuals who test positive. We suggest that trials are planned based on what is known about the test sensitivity and specificity in order to estimate the probability of having an event given test positive. One might also consider an adaptive trial design, with re-estimation of sample size based on data arising from the earliest recruits.

To help choose the most appropriate design, researchers can estimate “the proportion of participants dying from cancer who have a positive study sample” to consider using a targeted analysis over the traditional analysis, and “the probability of an individual with a positive test dying from cancer (during the study follow-up)” to consider the intended-effect analysis rather than the targeted analysis. A referee suggested that one might consider a hybrid design when not everyone is tested but a (stratified) sample of the control arm is taken in addition to all those individuals with an event. Without stratification, one must test a large proportion of the population to achieve meaningful gains over intended-effect analysis, making the rationale for (random) sampling weak compared with targeted or full intended-effect analysis. Further work to elucidate the potential benefit of stratified sampling and scenarios when it might be worthwhile would be helpful.

## Conclusion

Consideration of the mechanism by which cancer screening works leads us to recommend the following steps to improving the efficiencies of trials designed to study the efficacy of screening. First, we should aim to randomly assign individuals who attend a baseline study clinic and ideally only individuals who provide a baseline screening sample. Although this approach prevents estimation of the likely screening uptake or population-level impact, it will effectively eliminate noncompliance, may greatly reduce contamination, and hence increase the power to study screening efficacy.^11-13^ Second, where possible, we should collect samples from participants in the comparator arm to enable retrospective testing. By testing control samples from individuals who develop an event of interest, one not only greatly increases the power to detect the impact of intervening in individuals who screen positive but also gains insight into the causal pathways by which screening works. For example, when studying a test with a continuous outcome (such as quantitative fecal immunochemical testing or serum prostate-specific antigen testing), one can study the efficacy of using different thresholds, whereas a simple analysis of a 2-arm trial allows comparison only of 2 thresholds. Third, we have seen that the main assumptions needed to provide validity for retrospective testing analysis are (1) no effect of screening in individuals who (always) test negative, (2) no impact of storage on test results, and (3) independence between study arms and provision of samples before cancer diagnosis. Fourth, although there is some gain in power from testing all control samples, in many situations the gain will be small and not worth the cost or the complication of deciding whether to inform control individuals that an archival sample has tested positive. Fifth, where feasible, trials that follow our recommendations may be smaller and more precise than traditional trials. For example, secondary endpoints, such as overdiagnosis, may be estimated with much greater precision than the traditional approach. Finally, once a screening test has been demonstrated to show efficacy, randomized studies focusing on other aspects may be appropriate. For example, one might conduct (or embed within pilot studies) separate trials to compare different approaches within the subpopulations from Figure 1. These could include interventions to maximize screening uptake or different approaches to triage or evaluate risk of harm from revealing negative test results. Results from all these studies can be combined to model the overall effectiveness of an implemented screening program that may not be identical to that first studied in an efficacy trial.

## Supplementary Material

djae251_Supplementary_Data

## Data Availability

The data underlying this article are available in the article and in its online supplementary material.
