# Supplementary material for: More efficient, smaller multicancer screening trials
Source: J Natl Cancer Inst. 2024 Oct 14;117(3):450–5. doi: 10.1093/jnci/djae251 (PMC11884843; doi:10.1093/jnci/djae251)
Supplement: djae251_Supplementary_Data [file djae251_supplementary_data.pdf]

More Efficient Smaller Multi-Cancer Screening Trials: supplementary material

Peter Sasieni Ph.D. (1,*) and Adam R Brentnall Ph.D.(1)

(1) Wolfson Institute of Population Health, Queen Mary University of London

*Corresponding author, Wolfson Institute of Population Health, Queen Mary University of London, London, EC1M 6BQ, UK; p.sasieni@qmul.ac.uk

Supplementary Methods

Sample size

Set-up

We focus on analysis methods of a trial with randomisation in population B (see Figure 1 in the main paper). Let π be the proportion of those who provide a sample (population B) whose sample is positive (population C). The complement (B\C) is the population that only have test-negative samples. Denote by ρ_n the probability of an event (the risk) in population B\C (test negative) and ρ_p be the risk in population C (test positive). Let the (relative) benefit of screening positive be θ (note that this is not the efficacy of the cancer treatment per se, but the benefit of intervening (early) in those who are screen-positive). These parameters are summarised in Table S1.

Supplementary Table 1: Parameter summary

Parameter	Description
π	Proportion with a positive test result of those tested
θ	Relative risk in test positive individuals receiving triage (and treatment if required) relative to teh risk in test positive individuals who test result was not known (until after diagnosis)
ρ_n	Absolute risk in test-negative individuals
ρ_p	Absolute risk in test-positive individuals without intervention

Assumptions

1. No effect of testing on outcomes in those who test negative (population in B\C).
2. No effect of storage on test performance.
3. No difference in sample provision (in those without a previous positive sample) between arms (e.g., the probability of returning a sample at visit 3 in someone that has not had a previous sample that would have been positive if tested is independent of randomisation).

Approximate sample size formula

Suppose testing uses a chi-square test of proportions. Let p_1 and p_0 be the proportion with an event under the alternative hypothesis and n_1 and n_0 be the numbers randomised to the intervention and control arms, respectively. For an allocation ratio $R = n_1/n_0$, the pooled proportion is $p = (p_0 + Rp_1)/(1 + R)$. Sample size depends on the pooled variance $p(1 - p)(1/n_0 + 1/n_1)$ and the variance of the difference in proportions $p_0(1 - p_0)/n_0 + p_1(1 - p_1)/n_1$. If $p_1(1 - p_1)$ and $p_0(1 - p_0)$ are similar to each other then the

Supplementary Table 2: Event probabilities, difference and pooled variance of different analyses

Analysis group	Event probability			
	Intervention (p_1)	Control (p_0)	$p_1 - p_0$	Variance (pooled)
A1: Intended effect	$\theta\rho_p$	ρ_p	$(1-\theta)\rho_p$	$\theta^*\rho_p(1-\theta^*\rho_p) \approx \theta^*\rho_p$
A2: Targeted	$\pi\theta\rho_p$	$\pi\rho_p$	$(1-\theta)\rho_p\pi$	$\theta^*\pi\rho_p(1-\theta^*\pi\rho_p) \approx \pi\theta^*\rho_p$
A3. Traditional (all tested)	$p_{A31} = (1-\pi)\rho_n + \pi\theta\rho_p$	$p_{A30} = (1-\pi)\rho_n + \pi\rho_p$	$(1-\theta)\rho_p\pi$	$p_{A31*}(1 - p_{A31*}) \approx p_{A31*} = (1-\pi)\rho_n + \pi\theta^*\rho_p$

Footnote: A1: 'Test-positive' population C, endpoint cancer death; A2: 'Tested' population (B), endpoint 'test positive' cancer death; A3: 'Tested' population (B), endpoint cancer death. θ^* is between θ and 1 such that the risk with θ^* is roughly the pooled risk between the two arms of the study. p_{A31*} is the formula for p_1 in analysis group A3 when θ is replaced by $\theta*$.

total sample size $n = n_0 + n_1$ to achieve power β at the one-sided α level is approximately

$$\frac{(1+R)^2\{z_{1-\alpha} - z_\beta\}^2 p(1-p)}{R(p_1-p_0)^2} \quad = \quad KN,$$

say, where $K = (1+R)^2\{z_{1-\alpha} - z_\beta\}^2/R$, $N = p(1-p)/(p_1-p_0)^2$ and z_q is the q-th quantile of the standard normal distribution. Thus (for a given α, β, and R) the sample size is approximately proportional to the ratio of the pooled variance to the square of the effect size.

Comparison of sample size requirements

For different analysis groups, Table S2 provides mathematical expressions for the event probabilities (risk) in the two arms, the risk difference, and (half of the) approximate variance of the risk difference. These are applied next to different analysis methods assuming the risk of the event in the target population is small so that we can approximate the binomial variance $p(1-p)$ by the Poisson variance p except when restricted to test positive individuals (population C) when the proportion with an event *might* be substantial.

Intended-effect analysis

This is the 'test-positive' population (C), with endpoint cancer death. The proportion of test positive individuals dying from cancer in the each arm is $\theta\rho_p$ and ρ_p. Sample size is approximately proportional to $\theta^*\rho_p(1-\theta^*\rho_p)/\{(1-\theta)^2\rho_p^2\}$ for some θ^* between θ and 1 (such that the risk with θ^* is roughly the pooled risk between the two arms of the study; for approximations, we suggest replacing the unknown θ^* by $(1+R\theta)/(1+R)$ here and in the following formulae). In order to have that number of test-positive individuals, the expected number of participants required will be proportional to $N_0 = \theta^*\rho_p(1-\theta^*\rho_p)/\{\pi(1-\theta)^2\rho_p^2\}$ (i.e. divide by π).

Targeted analysis

This is the 'Tested' population (B), with endpoint 'test positive cancer death'. The proportion of individuals dying from cancer and with a positive test in each arm is $\alpha\theta\rho_p$ and $\alpha\rho_p$. So, the sample size is approximately proportional to $N_1 = \pi\theta^*\rho_p(1-\pi\theta^*\rho_p)/\pi^2(1-\theta)^2\rho_p^2 = N_0(1-\pi\theta^*\rho_p)/(1-\theta^*\rho_p) \approx N_0/(1-\theta^*\rho_p)$ (because $\pi\rho_p$ is small because it is smaller than the risk in the screened population B). Note that $(1-\theta^*\rho_p)$ is the probability of not dying from cancer (during follow-up) among test positive individuals (in the study as a whole). So, if risk in test positives is high, there is a considerable sample-size saving by testing all control samples and using the intended-effect analysis. But if most test-positive individuals do not have the event, there is little gained over the targeted approach.

Traditional approach in population B (all tested)

This is the 'Tested' population (B), with endpoint cancer death. The proportion of individuals with the event in the two arms is respectively $\pi\theta\rho_p + (1-\pi)\rho_n$ and $\pi\rho_p + (1-\pi)\rho_n$. The difference in proportions, $\pi(1-\theta)\rho_p$, is the same as in the targeted approach, but the variance is larger. Sample size is approximately proportional to $N_2 = \{\pi\theta^*\rho_p + (1-\pi)\rho_n\}/\{\pi^2(1-\theta)^2\rho_p^2\} = N_1[\{\pi\theta^*\rho_p + (1-\pi)\rho_n\}(\pi\theta^*\rho_p)]$.

Note that $\pi\theta^*\rho_p/\{\pi\bar{\theta}^*\rho_p + (1-\pi)\rho_n\}$ is the proportion of events that are in test-positive individuals (pooled across both arms of the study). Therefore, the lower the proportion of events in test-positive individuals, the greater the saving by restricting analysis to events in test-positive individuals. This is because noise comes from cancer deaths in test-negative individuals.
